# Smooth muscle cell phenotypic switching occurs independent of aortic dilation in bicuspid aortic valve-associated ascending aortas

**DOI:** 10.1371/journal.pone.0306515

**Published:** 2024-07-02

**Authors:** Brittany Balint, Inés García Lascurain Bernstorff, Tanja Schwab, Hans-Joachim Schäfers

**Affiliations:** Department of Thoracic and Cardiovascular Surgery, Saarland University Medical Center, Homburg, Saar, Germany; Università degli Studi di Milano, ITALY

## Abstract

**Background:**

Bicuspid aortic valves (BAV) are frequently associated with ascending aortic aneurysms. The etiology is incompletely understood, but genetic factors, in addition to flow perturbations, are likely involved. Since loss of contractility and elaboration of extracellular matrix in the vessel wall are features of BAV-associated aortopathy, phenotypic modulation of smooth muscle cells (SMCs) may play a role.

**Methods:**

Ascending aortic tissue was collected intra-operatively from 25 individuals with normal (i.e., tricuspid) aortic valves (TAV) and from 25 individuals with BAVs. For both TAV and BAV, 10 patients had non-dilated (ND) and 15 patients had dilated (D) aortas. SMCs were isolated and cultured from a subset of patients from each group. Aortic tissue and SMCs were fluorescently immunolabeled for SMC phenotypic markers (i.e., alpha-smooth muscle actin (ASMA, contractile), vimentin (synthetic) and p16^INK4a^ and p21^Cip1^ (senescence). SMCs were also analyzed for replicative senescence in culture.

**Results:**

In normal-sized and dilated BAV aortas, SMCs switched from the contractile state to either synthetic or senescent phenotypes, as observed by loss of ASMA (ND: *P* = 0.001, D: *P* = 0.002) and associated increases in vimentin (ND: *P* = 0.03, D: *P* = 0.004) or p16/p21 (ND: *P* = 0.03, D: *P*<0.0001) compared to TAV. Dilatation of the aorta exacerbated SMC phenotypic switching in both BAV and TAV aortas (all *P*<0.05). In SMCs cultured from normal and dilated aortas, those isolated from BAV reached replicative senescence faster than those from TAV aortas (all *P* = 0.02). Furthermore, there was a stark inverse correlation between ASMA and cell passage number in BAV SMCs (ND: *P* = 0.0006, D: *P* = 0.01), but not in TAV SMCs (ND: *P* = 0.93, D: *P* = 0.20).

**Conclusions:**

The findings of this study provide direct evidence from cell culture studies implying that SMCs switch from the contractile state to either synthetic or senescent phenotypes in the non-dilated BAV aorta. In cultured SMCs from both non-dilated and dilated aortas, we found that this process may precede dilatation and accompany aneurysm development in BAV. Our findings suggest that therapeutically targeting SMC phenotypic modulation in BAV patients may be a viable option to prevent or delay ascending aortic aneurysm formation.

## Introduction

Bicuspid aortic valves (BAVs) are frequently associated with dilatation of the ascending aorta [1,2]. The etiology of these aneurysms and the consecutive aortic complications is incompletely understood. Both genetic factors and blood flow turbulence have been implicated [3–6]. In BAV-associated ascending aortic aneurysms, common degenerative features are well-recognized. These include elastin degradation, accumulation of mucoid extracellular matrix (ECM) and smooth muscle cell (SMC) loss or modulation [7–10]. The underlying mechanisms are not well defined and, thus, therapeutic strategies are lacking.

Medial SMCs maintain structural integrity and ensure synchronized vascular tone of the aorta. This is achieved by coordinated layers of contractile SMCs circumferentially wrapping around the vessel wall, and forming strong attachments with each other and with the ECM [11]. Inherently abnormal SMCs can render the aortic wall vulnerable to dilation, dissection and rupture, as evidenced by a number of SMC-specific genetic mutations (i.e., MYH11, ASMA, MYLK) [12–14]. Normal, contractile SMCs can be reversibly modified to a dedifferentiated state wherein they exhibit a synthetic phenotype [15–17]. Synthetic SMCs are conceivably hazardous in the adult aorta, as they contribute to aortic degeneration through production of matrix metalloproteinases (MMPs) [8,18–20]. We recently showed that ascending aortic SMCs exhibit phenotypic switching in relation to increased patient age, despite having normal tricuspid aortic valve anatomy and normal ascending aortic dimensions [21]. Whether BAV anatomy is also related to SMC phenotypic modulation in the ascending aorta is less well-defined.

SMC abnormalities are also thought to play a role in BAV aortopathy. For instance, non-contractile, synthetic SMCs were derived from BAV-associated ascending aortic aneurysms [22]. SMCs in BAV aneurysms may also become senescent, an alternate phenotypic state that is characterized by essentially permanent cell cycle arrest with a shift in metabolic activity [23]. Previous findings identifying senescent SMCs in BAV aortic aneurysms showed that their senescence-associated secretory phenotype exhibited a propensity for favoring ECM degradation, with an increase in MMP expression [7]. Several lines of evidence suggest that there are distinct mechanisms of ascending aortic aneurysm development between BAV- and TAV-associated aortas [24–27]. These mechanistic differences could be due to distinctive genetic backgrounds, or due, in part, to incomplete penetrance of mutations that cause the BAV phenotype [6]. Whether unfavourable changes in SMC characteristics are specific to aortopathies associated with phenotypic BAVs is less known.

Understanding the underlying cellular mechanisms could stimulate the development of therapeutic strategies to prevent or delay aortic degeneration in individuals with BAVs. Therefore, we assessed for differences in SMC phenotypes in ascending aortic tissue and in cultured SMCs from patients with TAVs and BAVs. With the unique ability to compare non-dilated aortas from each group, we were able to better understand the impact of BAV morphology per se on SMC phenotypes, without secondary effects of aortic dilatation. We also compared aneurysmal aortas from both groups to determine whether dilatation exacerbates SMC phenotypic modulation. By studying isolated SMCs in culture, we were able to assess long-term changes in SMC phenotype with BAV versus TAV origin.

## Methods

The data presented in this manuscript are available in the (S1 File). This study complies with the Declaration of Helsinki, and was carried out with approval from the Saarland regional ethics committee (Ständige Ethikkommission der Ärztekammer des Saarlandes, Proposal # 47/14, Date of Issue: 12/08/2010). Written informed consent was obtained from all patients. Participants were prospectively recruited from December 1^st^ 2019 to December 1^st^ 2022.

### Patient enrollment

Ascending aortic tissue was extracted intra-operatively from 50 consecutive cardiac patients undergoing aortic valve or ascending aortic replacement surgery. Morphology of the aortic valve was determined pre-operatively by either trans-thoracic or trans-esophageal echocardiology, and was confirmed intra-operatively by the primary surgeon. Patients with normal (i.e., tricuspid (TAV)) aortic valves (n = 25) or with BAVs (n = 25) were enrolled. Aortic dimensions were determined by computed tomography prior to surgery, and they were confirmed intra-operatively via trans-esophageal echocardiography. Aortic diameters measuring ≥40mm were considered dilated [28]. For both the TAV and BAV groups, 10 patients had non-dilated (ND) aortas and 15 patients had dilated (D) aortas.

### Exclusion criteria

Patients were excluded if they had chronic viral diseases (i.e., HIV, Hepatitis B, Hepatitis C) or clinical symptoms of connective tissue disorders (e.g. Marfan syndrome, Loeys-Dietz syndrome). Aortic samples were macro- and micro- scopically examined, and those with evidence of inflammatory disease (or atherosclerosis) were excluded.

### Procurement of ascending aortic tissue

For each enrolled patient, a circumferential portion of aortic tissue (~4mm width) was excised from the anterior circumference of the thoracic aorta, 5-10mm above the sinotubular junction. Tissue was divided in the operating room; one fragment was immediately fixed in 4% phosphate-buffered formalin for histological studies. Formalin-fixed aortic tissue samples were embedded in paraffin, and then sectioned at 1μm thickness. The remaining fresh aortic fragment was transferred in PBS to a sterile tissue culture hood for instant isolation of medial SMCs (TAV: ND = 8, D = 5; BAV: ND = 7, D = 5).

### SMC isolation and culture

SMCs were isolated from the medial layer of non-dilated and dilated (≥40mm) aortic tissue samples through enzymatic digestion with a purified collagenase blend (Liberase^TM^), as previously described [21]. Briefly, the medial layer was separated by gently scraping away the intimal layer with a scalpel, and by peeling away the adventitial layer with forceps. The medial layer was then cut into small pieces (~1x1mm), which were added to the digestion media (830μl of M199 Media (ThermoFisher Scientific, 11150059; + 0.5% FBS + 1% penicillin/streptomycin), 150μl of Liberase^TM^ (Roche, 05401020001) and 30μl of DNAse) for 2 rounds of 2h incubation periods at 37°C, with refreshed digestion media between incubation rounds. Isolated SMCs were plated onto 60mm cell culture dishes, and maintained in optimized culture conditions (37°C, 5% CO_2_, and 95% humidity), and media (M199 media, ThermoFisher Scientific, 11150059; + 10% FBS + 1% penicillin/streptomycin) was changed every 48h until confluence was reached. At ~95% confluence, SMCs were chemically detached from the culture dishes (Trypsin/EDTA solution, ThermoFisher Scientific, R001100) and were either re-plated on coverslips for immunocytochemistry (cell passage 1), or they were re-plated on fresh gelatin-coated tissue culture dishes for replicative capacity assessment. SMCs were repeatedly re-plated until growth was arrested and cells no longer achieved confluence. The maximum cell passage of each SMC culture was considered the replicative capacity. For immunocytochemistry, the SMCs were grown on coverslips until 80% confluence was reached, at which point they were serum-starved (0.5% FBS) for 72h. Serum-starved SMCs were then fixed with 4% paraformaldehyde (cell passage 1).

### Immunostaining of aortic tissue and cultured SMCs

Formalin-fixed paraffin-embedded aortic tissue sections and paraformaldehyde-fixed aortic SMCs were immunolabeled for markers of vascular SMC phenotypes. We utilized rabbit polyclonal antibodies targeted against the SMC-specific contractile protein, alpha-smooth muscle actin [19] (ASMA; 1:100, ab5694, Abcam), or against vimentin (1:100, ab137321, Abcam), which is abundantly expressed in synthetic SMCs [29]. Cellular senescence was evaluated by immunolabeling for p16^INK4a^ (monoclonal mouse, 1:50, MA5-17054, Invitrogen) and p21^Cip1^ (1:50, MA1-33926, Invitrogen), cyclin-dependent kinase inhibitors that delineate two core senescence pathways [23,30]. Bound primary antibodies were visualized with Alexa-594-conjugated secondary goat anti-rabbit or anti-mouse antibodies. DAPI was used to counterstain and label cell nuclei in aortic tissue samples, which were then mounted onto microscopy slides. Coverslips with immunolabeled SMCs were affixed onto microscopy slides with DAPI-containing mounting media (Vectashield®, H-1200-10).

### Fluorescence microscopy and image analysis

Images of fluorescently-immunolabeled aortic tissue and SMCs were captured using a laser scanning confocal microscope (Zeiss LSM, Plan Apochromat). For each stain, 10 regions of interest were captured per patient for both aortic tissue (40x, 1.3 oil objective) and SMCs (20x, 0.8 M27 objective). Image acquisition and analyses were performed by 2 blinded evaluators. For ASMA and vimentin, the fluorescent intensity of each stain was measured with ImageJ (NIH). Mean fluorescent intensity of each image was normalized to the background fluorescent intensity. p16^INK4a^ and p21^Cip1^ positivity were measured by calculating the percentage of positively-labeled SMC nuclei in each region of interest.

### Statistics

All statistical analyses were carried out using Prism 9 (Graphpad Software). Prior to group comparisons, datasets were tested for normality using the D’Agostino and Pearson omnibus test. When both datasets were normally distributed, the Student’s t-test was used to make comparisons between groups. If one or both data sets failed the normality test, data sets were compared with the Mann-Whitney U test. Relationships between continuous variables were assessed by linear regression analyses. Patient age data are presented as mean ± standard deviation. Statistical significance was set at *P*<0.05.

### Data availability/availability of data and materials

The datasets used and/or analysed during the current study are available in the in the (S1 File).

## Results

### Patient characteristics

Ascending aortic tissue was extracted intra-operatively from patients with either TAVs (n = 25) or BAVs (n = 25). Of the TAV patients, 10 had non-dilated aortas (mean age: 59.1±9.3, range: 24–72 years) and 15 had aneurysmal aortas (mean age: 54.9±11.7, range: 24–73 years). Similarly, 10 BAV patients had normal-sized aortas (mean age: 42.3±14.4, range: 15–64 years) and 15 had aneurysmal aortas (mean age: 49.2±10.3, rage: 20–81). Patient age between groups only differed in the BAV ND group, *which was significantly younger* than all other groups (*P* = 0.01). Co-morbidities, including hypertension and smoking, were similar between groups (all *P*>0.05. Furthermore, prescribed medications did not differ between groups (all *P*>0.05). Clinical characteristics of all enrolled patients are presented in **Table 1**. Vascular SMCs were extracted from the medial layer of aortic tissue from patients in each group. Due to tissue availability, SMCs were isolated from 13 TAV (ND = 8, D = 5) and 12 BAV (ND = 7, D = 5) patients. Patients from the cell culture BAV ND and BAV D groups were significantly younger than those from the TAV ND (*P* = 0.02) and TAV D (*P* = 0.04) groups, respectively.

**Table 1:** Patient characteristics.

	**TAV**		**BAV**	
	Normal	Dilated	Normal	Dilated
# of Patients	10	15	10	15
Patient Age (Range)	24–72	28–73	15–64	20–81
Patient Age (Mean±SD)	59.1±9.3	54.9±11.7	42.3±14.4*	49.2±10.3
Ascending Aortic Diameter (Range; cm)	2.5–3.9	4.1–5.2	2.4–4.0	4.1–5.6
Ascending Aortic Diameter (Mean±SD)	3.1±0.4	4.6±0.3†	3.2±0.5	4.7±0.4†
	Co-Morbidities			
Hypertension (%)	3 (30)	4 (26.7)	4 (40)	5 (33.3)
Hyperlipidemia (%)	2 (20)	3 (20)	3 (30)	2 (13.3)
Smoker (%)	1 (10)	2 (13.3)	0 (0)	0 (0)
Diabetes (%)	1 (10)	1 (6.6)	0 (0)	1 (6.6)
	Medications			
ß-Blocker (%)	2 (20)	4 (26.7)	2 (20)	3 (20)
ACE Inhibitor (%)	1 (10)	1 (6.6)	1 (10)	2 (13.3)
Diuretic (%)	1 (10)	2 (13.3)	4 (11)	1 (10)
Calcium Channel Blocker (%)	2 (20)	1 (6.6)	5 (14)	3 (20)
Statin (%)	2 (20)	2 (13.3)	3 (9)	2 (13.3)

Patient characteristics, co-morbidities and medications for each group, based on aortic valve morphology (TAV and BAV) and dilatation status (Normal, Dilated).

*P<0.05 versus TAV Normal.

†P<0.05 versus Normal dilatation status group for each aortic valve morphology group.

### Phenotypic switching of SMCs in non-dilated BAV aortas

**Fig 1:**
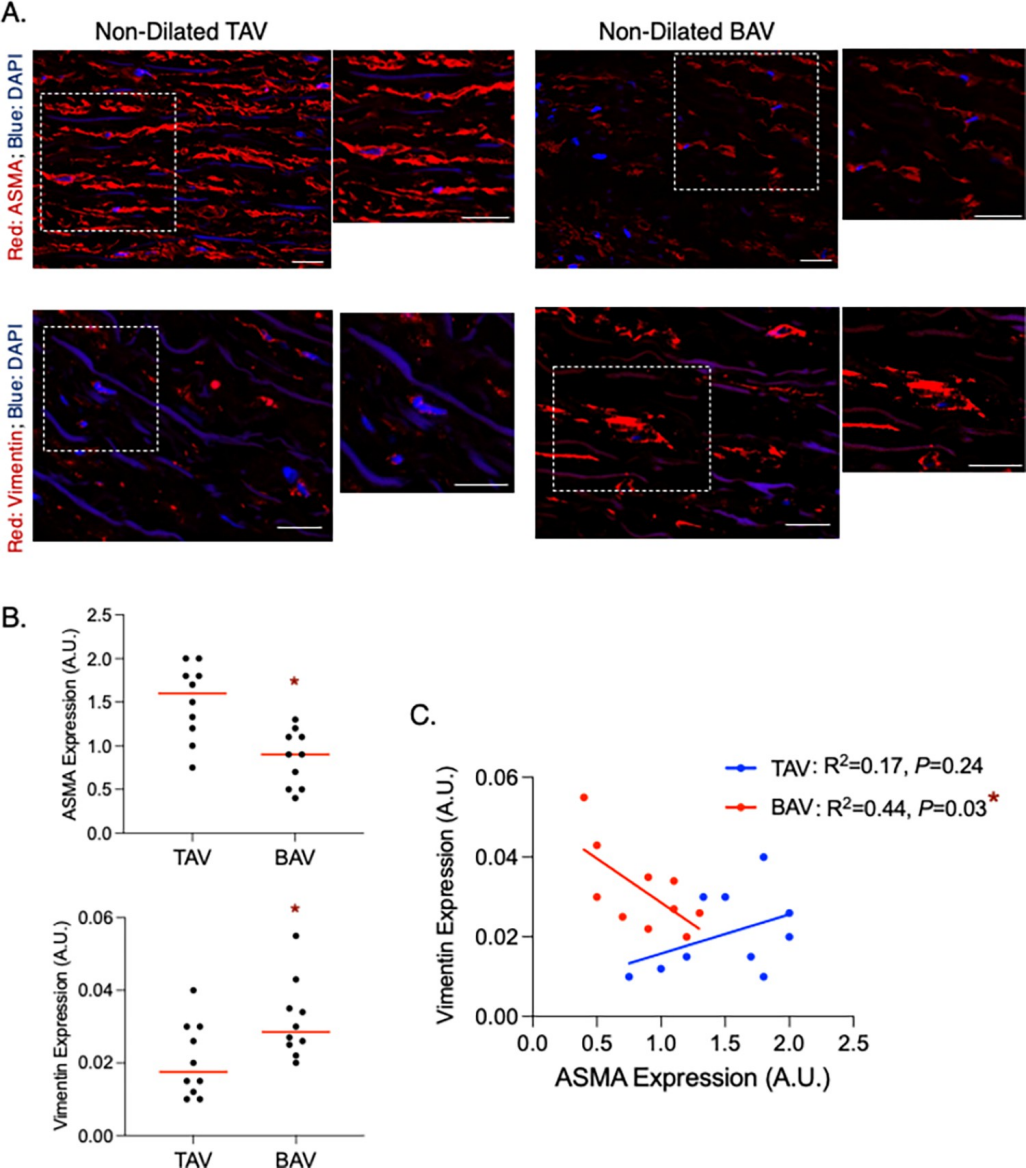
Alpha-smooth muscle actin (ASMA) is decreased while vimentin is increased in non-dilated bicuspid aortic valve (BAV)-associated aortas. **A.** Fluorescent micrographs of ASMA (top) and vimentin (bottom) in non-dilated aortic tissue from individuals with normal (i.e., tricuspid) aortic valves (TAV; left) and BAVs (right). Higher magnification images of the boxed region are shown to the right of each image. **B.** Graphs depicting ASMA (top) and vimentin (bottom) expression in the normal ascending aorta from TAV (N = 10) and BAV (N = 10) patients. Horizontal bars represent median values. **C.** Graph depicting the relationship between ASMA and vimentin expression in the non-dilated aorta of TAV (N = 10) and BAV (N = 10) patients. A.U. = arbitrary units; * = statistical significance; scale bar = 25 μm.

Analysis of phenotypic markers in non-dilated aortic tissue revealed a decrease in ASMA in BAV aortas compared to TAV (*P* = 0.001, **Fig 1A and 1B**). Furthermore, vimentin levels were significantly increased in BAV non-dilated aortas (*P* = 0.03, **Fig 1A and 1B**). Linear regression analysis revealed an inverse relationship between ASMA and vimentin in BAV ND aortas (R^2^ = 0.44, *P* = 0.04), but not in TAV ND aortas (R^2^ = 0.17, *P* = 0.24), which denotes a switch from contractile to synthetic SMCs in normal-sized BAV aortas (**Fig 1C)**.

**Fig 2:**
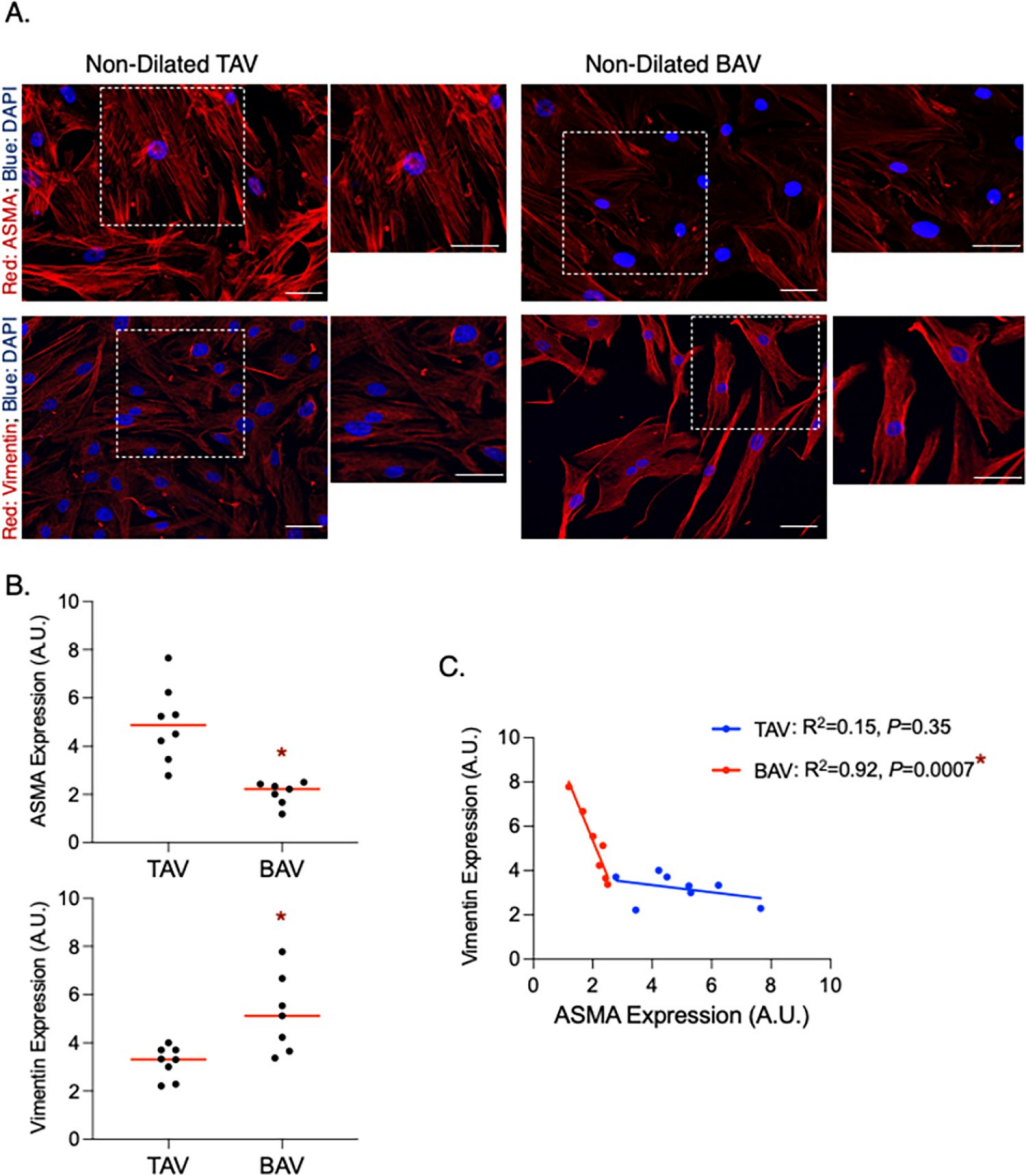
Alpha-smooth muscle actin (ASMA) is decreased and vimentin is increased in smooth muscle cells (SMCs) isolated from non-dilated bicuspid aortic valve (BAV)-associated aortas. **A.** Fluorescent micrographs of ASMA (top) and vimentin (bottom) in SMCs isolated from non-dilated aortas associated with normal (i.e., tricuspid) aortic valves (TAV; left) and BAVs (right). Higher magnification images of the boxed region are shown to the right of each image. **B.** Graphs depicting ASMA (top) and vimentin (bottom) expression in SMCs from from each group (TAV: N = 8, BAV: N = 7). Horizontal bars represent median values. **C.** Graph depicting the relationship between ASMA and vimentin in SMCs for each group (TAV: N = 8, BAV: N = 7). A.U. = arbitrary units; * = statistical significance; Scale bar = 20 μm.

By isolating SMCs from non-dilated TAV and BAV aortas, we confirmed that our findings are SMC-specific. In SMCs isolated from BAV aortas, there was a decrease in ASMA (*P* = 0.0004) and an increase in vimentin (*P* = 0.007) compared to TAV ND SMCs (**Fig 2A and 2B**). As in aortic tissue, there was an inverse linear relationship between ASMA and vimentin levels in SMCs from individual BAV patients (R^2^ = 0.92, *P* = 0.0007; **Fig 2C**). Conversely, a relationship between ASMA and vimentin concentration was not observed in TAV ND SMCs (R^2^ = 0.15, *P* = 0.35; **Fig 2C**).

### SMCs switch from the contractile to the synthetic phenotype in dilated aortas

**Fig 3:**
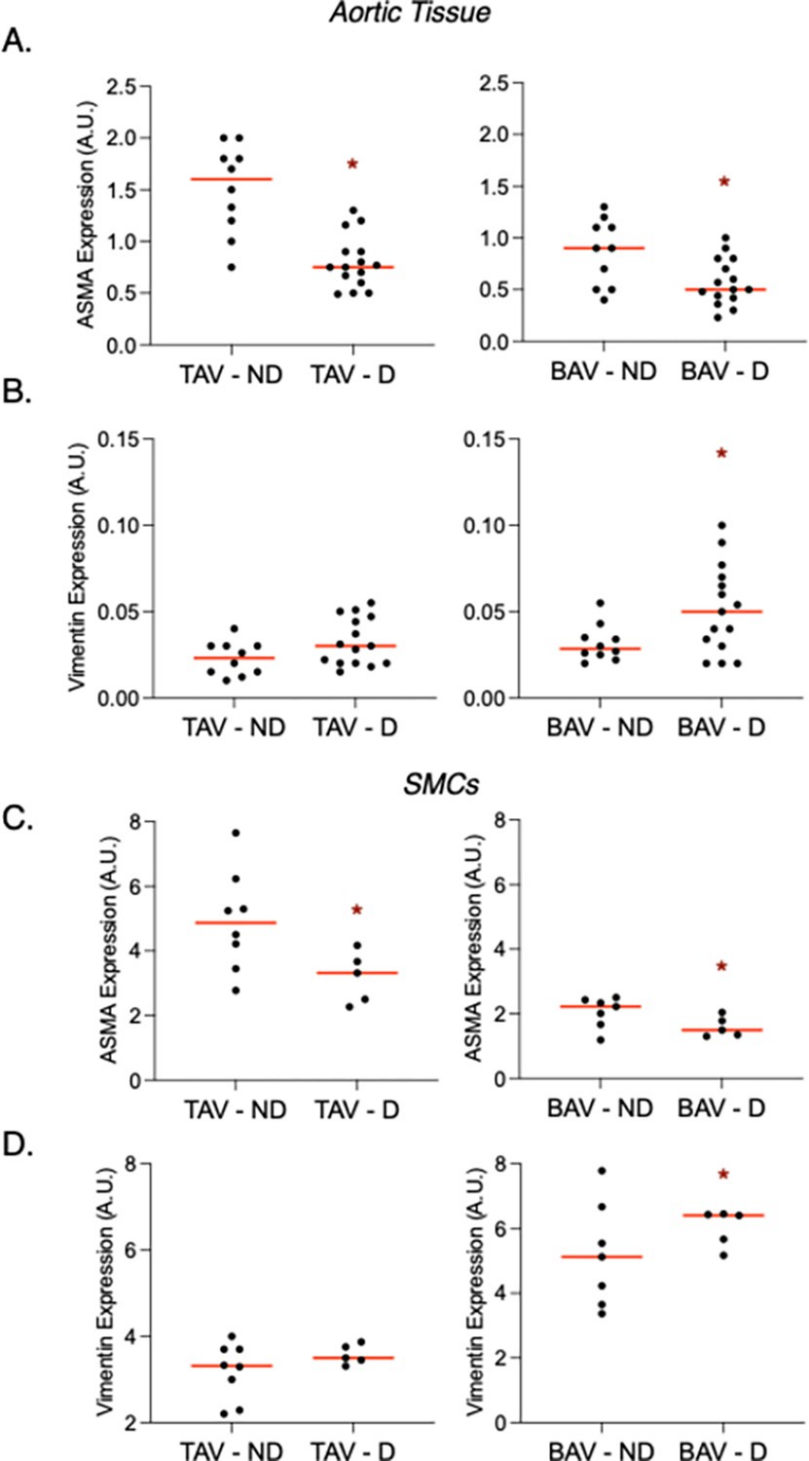
Dilatation of the ascending aorta impacts alpha-smooth muscle actin (ASMA) and vimentin expression in bicuspid aortic valve (BAV)-associated aortas and smooth muscle cells (SMCs). Graphs depicting ASMA (**A**) and vimentin (**B**) expression in non-dilated (ND) and dilated (D) ascending aortic tissue from tricuspid aortic valve (TAV; left)- and BAV (right)- associated aortas. Graphs depicting ASMA (**C**) and vimentin (**D**) expression in SMCs isolated from ND and D aortas from TAV (left) and BAV (right) patients. Horizontal bars represent median values. * = statistical significance.

While assessing the impact of dilatation, we found that in D BAV aortas, ASMA was decreased (*P* = 0.01) and vimentin was increased (*P* = 0.03) compared to ND BAV aortas (**Fig 3A and 3B)**. For TAV, ASMA was also decreased in D versus ND aortas (*P*<0.0001), but vimentin levels were statistically similar (*P* = 0.07; **Fig 3A and 3B**). Comparable results were observed in isolated SMCs, as ASMA was increased and vimentin was decreased in D versus ND BAV SMCs (all *P* = 0.04; **Fig 3C and 3D**). Furthermore, ASMA was decreased in TAV D versus ND SMCs (*P* = 0.04) while vimentin levels remained similar (*P* = 0.23; **Fig 3C and 3D**).

**Fig 4:**
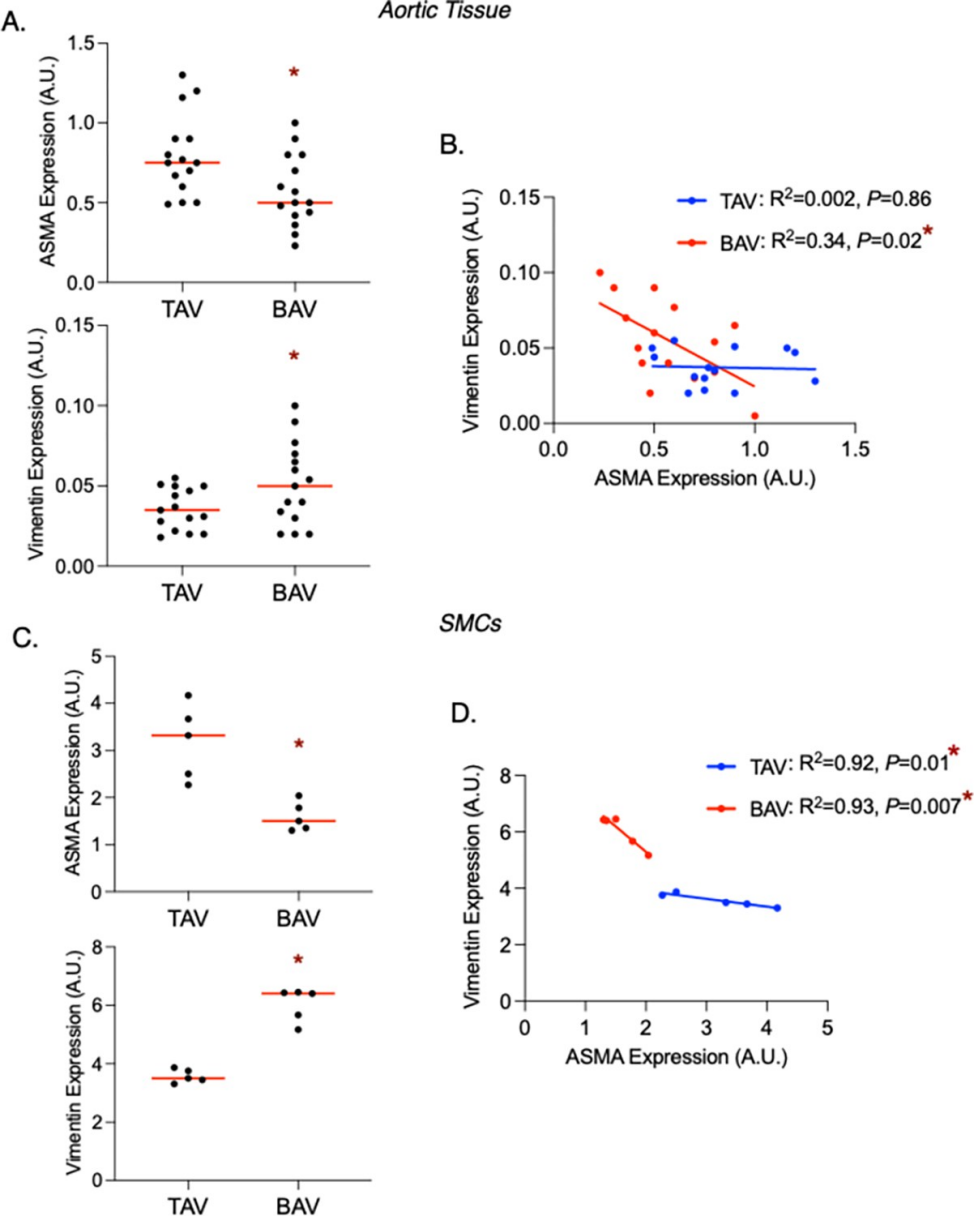
Alpha-smooth muscle actin (ASMA) is decreased and vimentin is increased in dilated bicuspid aortic valve (BAV)-associated aortas. **A,C.** Graphs depicting ASMA (top) and vimentin (bottom) in dilated aortic tissue (**A**) and smooth muscle cells (SMCs, **C**) from individuals tricuspid aortic valves (TAV) and BAVs. **B,D.** Graph depicting relationships between ASMA and vimentin expression in the dilated aorta (**B**) or isolated SMCs (**D**) from TAV and BAV patients. Horizontal bars represent median values. A.U. = arbitrary units; * = statistical significance.

Since dilatation impacted ASMA levels in both TAV and BAV aortas, we asked whether differences in SMC phenotypes can be observed between TAV and BAV aneurysmal aortas. In dilated aortic tissue, ASMA was decreased and vimentin was increased in BAV versus TAV (*P* = 0.02 and 0.04, respectively; **Fig 4A**). There was an inverse correlation between ASMA and vimentin for dilated BAV aortas (R^2^ = 0.34 *P* = 0.02), but not for dilated TAV aortas R^2^ = 0.002 *P* = 0.86; **Fig 4B)**. Phenotypic changes were retained in cultured SMCs, as ASMA was decreased and vimentin was increased in BAV versus TAV (*P* = 0.003 and 0.0004, respectively; **Fig 4C**). Like in SMCs from non-dilated aortas, there was an inverse relationship between ASMA and vimentin in BAV SMCs from dilated aortas (R^2^ = 0.93 *P* = 0.007; **Fig 4D**). In contrast to what we observed in normal-sized TAV aortas, there was a significant inverse relationship between ASMA and vimentin in TAV SMCs from aneurysmal aortas (R^2^ = 0.92 *P* = 0.01; **Fig 4D**).

### SMC senescence

**Fig 5:**
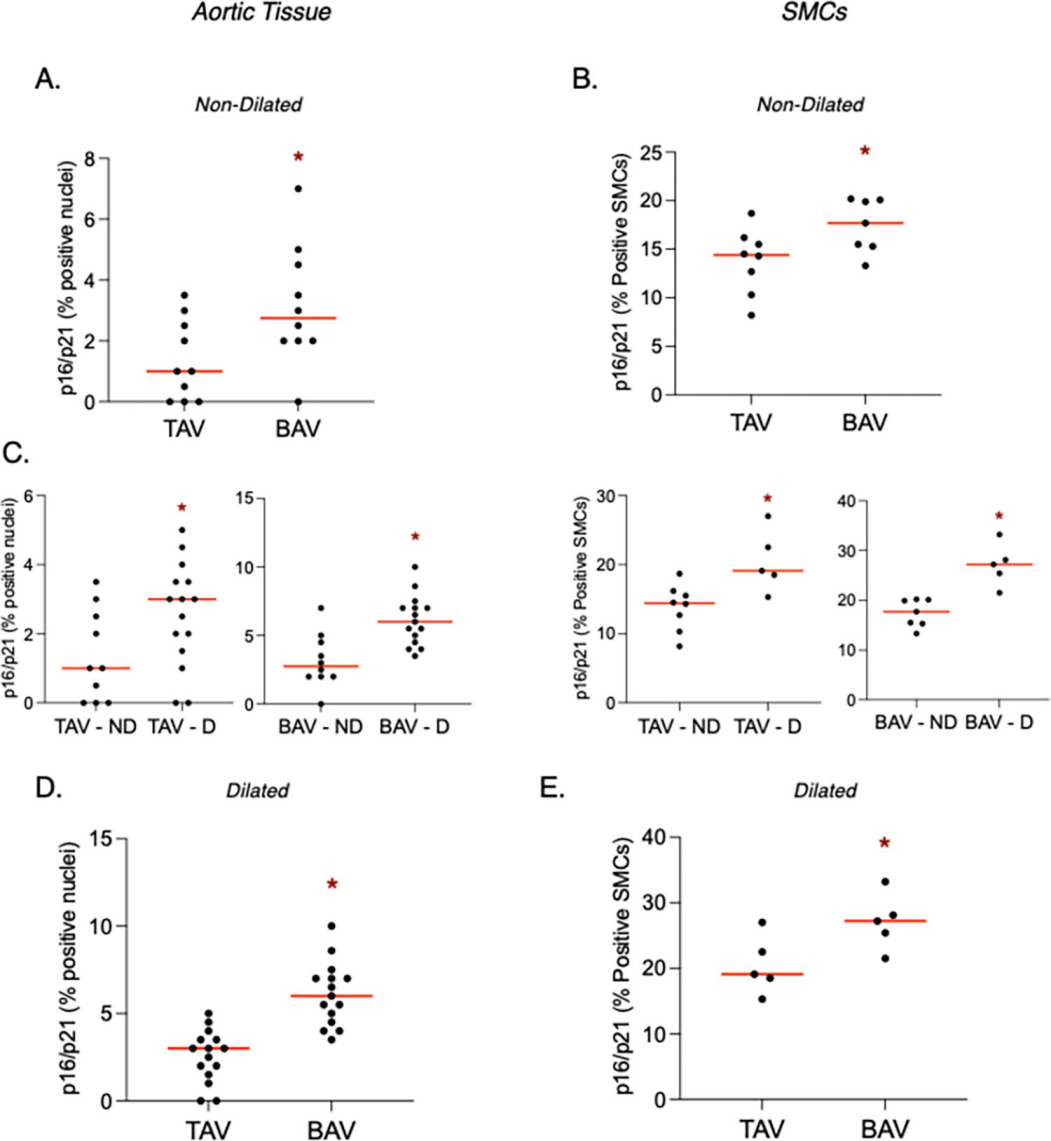
**Cell cycle inhibitors p16 and p21 are increased in the dilated aorta and in association with bicuspid aortic valves (BAV). A-B**. Graphs depicting p16/p21 positivity in the non-dilated aorta (**A**) and isolated smooth muscle cells (SMCs, **B**) in individuals with normal (i.e., tricuspid) aortic valves (TAV) or BAVs. **C.** Graphs depicting p16/p21 positivity in response to aortic dilatation (D) versus non-dilated (ND) in aortic tissue (left) and SMCs (right) from individuals with TAVs and BAVs. **D-E.** Graphs depicting p16/p21 positivity in the dilated aorta (**D**) and isolated SMCs (**E**) in individuals with TAVs or BAVs. Horizontal bars represent median values. * = statistical significance.

To evaluate SMC senescence in the ascending aorta and in isolated SMCs, we assessed for cell-cycle inhibitors p16^INK4a^ and p21^Cip1^. In BAV non-dilated aortas, we observed increased p16/p21 positivity in the medial layer compared to in TAV non-dilated aortas (*P* = 0.03; **Fig 5A**). Likewise in cultured SMCs, p16/p21 positivity was increased in SMCs from BAV versus TAV non-dilated aortas (*P* = 0.04; **Fig 5B**). In tissue and in isolated SMCs, p16/p21 positivity increased in response to dilatation for both TAV (*P* = 0.04 and 0.01, respectively) and BAV (*P* = 0.008 and 0.0007, respectively; **Fig 5C**). We, therefore, looked for differences in SMC senescence between TAV and BAV aneurysmal aortas, and found increased p16/p21 in BAV versus TAV dilated aortas (*P*<0.0001) and in SMCs isolated from dilated aortas (*P* = 0.04; **Fig 5D and 5E**).

### Phenotypic switching from contractile to senescent SMCs from BAV aortas

**Fig 6:**
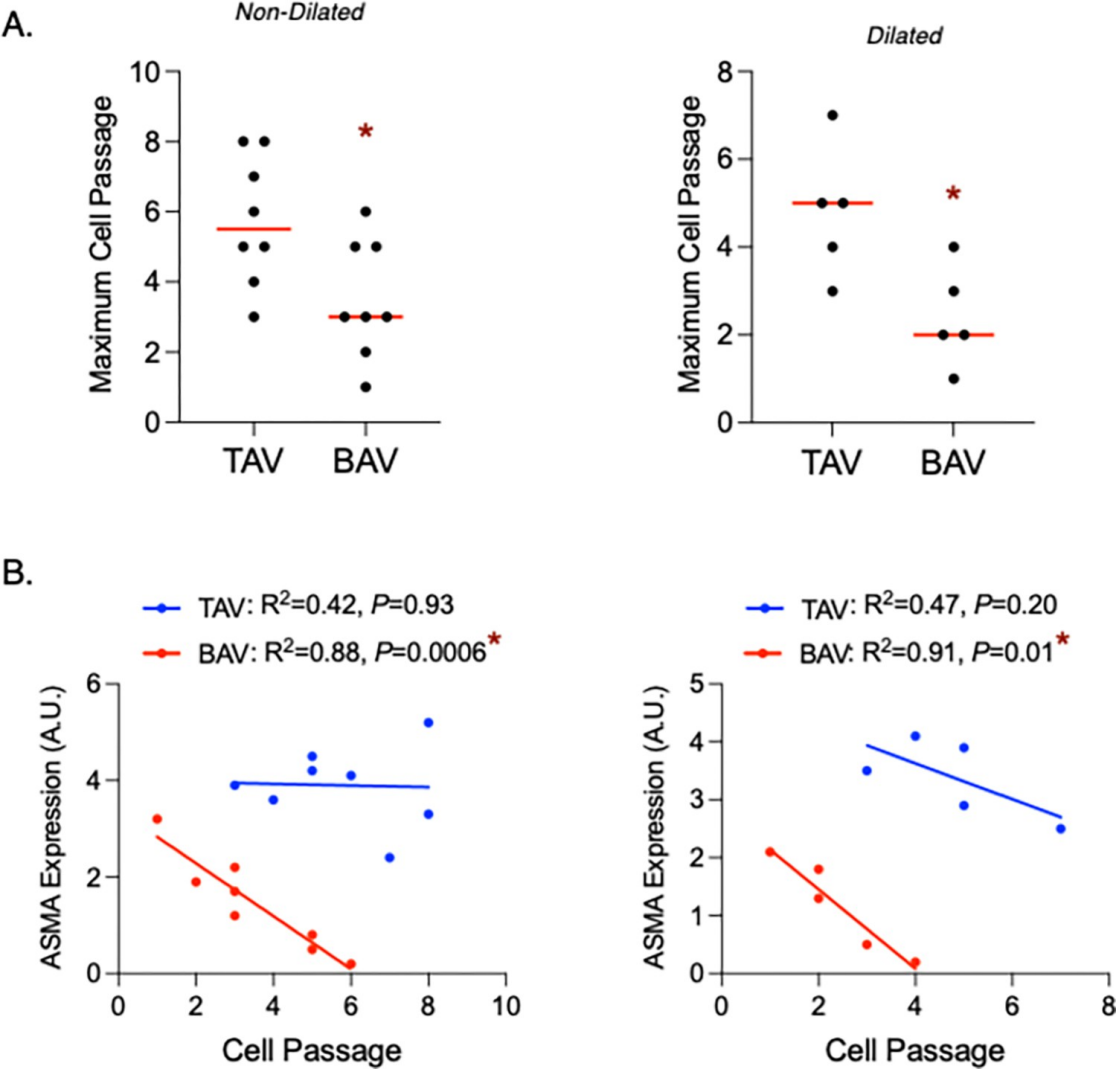
Smooth muscle cells (SMCs) from bicuspid aortic valve (BAV) aortas switch from contractile to senescent states over time in culture. **A**. Graphs depicting the maximum cell passage reached by SMCs isolated from non-dilated (left) or dilated aortas, from individuals with tricuspid aortic valves (TAVs) or BAVs. **B.** Graphs depicting the relationship between cell passage and ASMA levels in SMCs isolated from normal-sized (left) and dilated (right) TAV and BAV aortas. Horizontal bars represent median values. * = statistical significance. A.U. = arbitrary units.

By assessing the replicative capacity of cultured SMCs, we found that BAV SMCs enter replicative senescence earlier than TAV SMCs, both when comparing those isolated from non-dilated and dilated aortas (all *P* = 0.02, **Fig 6A)**. In order to establish direct evidence of phenotypic switching from contractile to senescent states, we analyzed ASMA levels in cultured SMCs at each cell passage leading up to replicative senescence. In TAV SMCs, there was no significant relationship between ASMA expression and cell passage (ND: R^2^ = 0.42, *P* = 0.93, D: R^2^ = 0.47, *P* = 0.20; **Fig 6B**). For BAV, however, there was a significant inverse relationship between ASMA and cell passage in SMCs from both normal-sized (ND: R^2^ = 0.88, *P* = 0.0006) and dilated aortas (D: R^2^ = 0.91, *P* = 0.01), with ASMA levels dropping to nearly zero at replicative senescence (**Fig 6B**). These results suggest that BAV SMCs switch from normal, contractile SMCs to the senescent state prematurely, even prior to dilatation.

## Discussion

It has long been recognized that SMCs play a role in vascular remodeling and aneurysm formation. The underlying mechanisms, however, are unclear. Compared with TAV, BAV-associated aortas have a higher progression rate of expansion [31,32], suggesting different mechanisms of thoracic aortic dilatation. Understanding the mechanistic differences between TAV and BAV-associated ascending aortic aneurysms could drive advances in personalized medicine and more precise treatment options. Therefore, we analyzed SMCs from TAV and BAV aneurysms with specific emphasis on phenotypic switching from healthy contractile to either synthetic or senescent phenotypes.

In aneurysmal ascending aortic tissue, we found that SMCs in BAV aortas are in a less differentiated state compared to those from TAV aortas. This is illustrated by a decrease in ASMA and an increase in vimentin concentrations in medial SMCs. These findings are consistent with a previous study in which ASMA expression was decreased in the aortic wall of BAV patients, both in dilated and non-dilated samples, compared to TAV counterparts [33]. For SMCs isolated from individual BAV patients, we found a direct inverse relationship between ASMA and vimentin concentrations, suggesting that contractile SMCs are replaced by synthetic SMCs in the BAV ascending aorta. This modulation of SMC phenotypes may be responsible, at least in part, for ECM remodeling that is consistently observed in BAV aortas, as synthetic SMCs that express high levels of vimentin secrete ECM-degrading enzymes, such as MMP-2 [34].

Recent data from single cell ATAC-seq analyses showed that stress-induced chromatin remodeling may be a potential mechanism of contractile gene suppression in human aortic SMCs. The observed epigenetic modifications in SMC contractile genes were attributed to mechanisms triggered by the accumulation of cytosolic DNA, which occurs due to stress-induced DNA damage [35]. Activation of the cytosolic DNA sensor STING led to subsequent activation of IRF3, thereby facilitating phenotypic alteration by initiating the upregulation of inflammatory gene expression and orchestrating EZH2-mediated epigenetic repression of SMC contractile gene expression [36]. This points to epigenetic factors playing a major role in the suppression of SMC contractile genes, and the induction of alternative phenotypes. Given the prevailing characterization of BAV aortopathy as predominantly featuring low inflammatory activity [37], further investigation is warranted to elucidate the epigenetic mechanisms underlying the induction of proliferative, migratory, and senescent cellular phenotypes in BAV aortic tissue.

Senescence is another SMC phenotype that has the potential to negatively impact the integrity of the aortic wall. SMCs enter senescence, a state of irreversible cell cycle arrest, as a protective mechanism when faced with overwhelming injury-triggers, such as unrepaired DNA damage [30]. Although senescence permits cell viability, it entails a shift in the secretory phenotype that promotes ECM degradation [7,23]. SMC senescence has been observed in aneurysmal thoracic aortas in mice [38] and humans, where ECM degradation was observed in the absence of inflammatory cytokines [7]. Here, we similarly showed that in aneurysmal aortic tissue and isolated SMCs, p16 and p21 are increased in BAV. We also found that the replicative capacity of SMCs was decreased in BAV, suggesting that BAV SMCs reach senescence earlier than those from TAV. Our findings are consistent with a previous study that found other senescence genes (i.e., MORC3, CDKN2A and MAP2K3) up-regulated in SMCs isolated from BAV aneurysmal thoracic aortas in connection with the DNA damage response [39], suggesting a role for SMC senescence in BAV aortopathy. Our data suggest a direct shift from contractile SMCs to senescent SMCs in the BAV aorta, as SMCs isolated from BAV aneurysms showed an inverse relationship between senescence markers and ASMA. Furthermore, ASMA concentration significantly decreased with increased cell passage, indicating a switch from contractile to senescent SMCs in BAV aortas that accumulates over time in culture. We also found a direct correlation between senescent and synthetic (vimentin) markers in BAV SMCs, implying that contractile SMCs either switch to the synthetic or senescent phenotype in BAV aneurysms. The mechanisms dictating whether a SMC enters a synthetic or senescent state in BAV aortas, however, should be further evaluated. Moreover, it warrants investigation whether epigenetic alterations initiated by stress-induced DNA damage, irrespective of concurrent alterations in inflammatory pathways, contribute to senescence in SMCs associated with bicuspid aortic valve BAV pathology. It is important to note that while our study employed established markers of cellular senescence, such as p16 and p21 expression levels, and assessed replicative capacity as an indicator of senescence, live-cell assays (i.e., senescence-associated beta-galactosidase) should be utilized in future studies as a more robust indicator of SMC senescence in the BAV ascending aorta.

SMC phenotypic modulation can be triggered by local injury and remodeling [16], consequences tightly linked to aortic dilatation. To assess whether SMC changes can precede dilatation and not simply occur as a result of dilatation-associated remodeling, we also studied SMCs from normal-sized aortas. Despite being significantly younger in age, a similar phenotypic shift of SMCs was observed in non-dilated BAV aortas, including decreased ASMA and increased vimentin and p16/p21 levels compared to TAV counterparts. Phenotypic switching of SMCs from contractile to non-contractile states could underlie aneurysm formation in BAV aortas, as loss-of-function mutations in ASMA are known to cause thoracic aortic aneurysms [12] due, in part, to their loss of contractility [40]. As with aneurysmal aortas, we observed inverse relationships between ASMA and both vimentin and p16/p21 levels in non-dilated aortas, providing evidence for SMC phenotypic switching to synthetic or senescent states in non-dilated BAV aortas. This is in line with an earlier study showing a significant reduction in ASMA and an increase in synthetic SMC markers prior to abdominal aneurysm formation in a mouse study [18]. While the mechanisms driving aortic dilatation may vary across different segments of the aorta, the identification of SMC phenotypic switching as a consistent phenomenon underscores its pathogenic significance in this process. Further studies assessing the relationship between phenotype markers in BAV SMCs from the ascending aorta are needed. Importantly, as the non-dilated BAV group was significantly younger in age, these findings suggest that the presence of a BAV may be more hazardous than patient age, which we recently showed to contribute to aberrant SMC phenotypic modulation [21]. In a study of human BAV ascending aortas, senescent SMCs were also observed prior to dilatation, and they were localized to regions of the aortic wall with obvious collagen degradation [7]. Taken together, our findings suggest that SMC phenotypic modulation occurs prior to aneurysm formation in BAV aortas, and may contribute to ECM degradation preceding aortic dilatation.

In summary, our findings suggest that SMC phenotypic modulation is a feature of BAV aortopathy. This is in accordance with prior research, where there is consensus that the transition of SMC phenotypes plays a pivotal role in vascular impairment, thereby contributing to the development of aortic aneurysms [7,18,21,23,41,42]. For the first time, we provide direct evidence from cell culture studies suggesting that contractile SMCs are replaced by either synthetic or senescent SMCs in the BAV aorta. Non-dilated aortic SMCs revealed that this process may precede aneurysm formation. By assessing primary SMCs in culture, we were able to assess SMC changes over time, revealing heightened senescence in BAV aortic SMCs compared to TAVs. Our findings suggest that therapeutics targeting SMC phenotypic modulation in BAV patients may be considered to prevent or delay ascending aortic aneurysm formation. Furthermore, these therapies should be considered at an earlier age in individuals with a BAV, as the presence of a BAV may override age-dependent SMC phenotypic switching. The mechanisms by which SMCs become synthetic versus senescent in BAV aortas should be evaluated to further enhance personalized medicine therapies in individuals with BAV.

## Supporting information

S1 File Data underlying findings described in manuscript. (XLSX)
